# C1 Facetectomy and Ventral Fixation of Occipitoatlantoaxial Complex for Concurrent Congenital Atlanto-Occipital Dislocation and Atlantoaxial Instability in a Toy Poodle

**DOI:** 10.3390/ani14131886

**Published:** 2024-06-26

**Authors:** Kyung-Bin Kim, Jae-Min Jeong, Young-Jin Jeon, Seong-Mok Jeong, Dae-Hyun Kim, Hae-Beom Lee

**Affiliations:** Department of Veterinary Surgery, College of Veterinary Medicine, Chungnam National University, 99, Daehak-ro, Yuseong-gu, Daejeon 34134, Republic of Korea; robin627@naver.com (K.-B.K.); klmie800@cnu.ac.kr (J.-M.J.); orangee0115@gmail.com (Y.-J.J.); jsmok@cnu.ac.kr (S.-M.J.); vet1982@cnu.ac.kr (D.-H.K.)

**Keywords:** atlanto-occipital dislocation, atlantoaxial instability, facetectomy, occipitoatlantoaxial, cranio-cervial junction disorders, dog

## Abstract

**Simple Summary:**

This case report details the treatment of a dog diagnosed with concurrent congenital atlanto-occipital dislocation and atlantoaxial instability, a rare condition in dogs. The surgical approach, which had not been previously reported for such cases, involved ventral facetectomy and stabilization using screws, wire, and polymethyl methacrylate from the occipital bone to C2. This novel procedure provided valuable insights into the management of complex cranio-cervical junction disorders in veterinary neurosurgery.

**Abstract:**

An 8-month-old, 3.4 kg, castrated male Toy Poodle was referred for progressive tetraparesis and respiratory disorder without a history of trauma. Repeated computed tomography (CT) and magnetic resonance imaging (MRI) with different positions of the neck revealed concurrent atlanto-occipital dislocation (AOD) and atlantoaxial instability (AAI) with spinal cord compression. This case was unique due to its congenital nature and the absence of trauma. The surgical treatment involved precise removal of the C1 vertebra’s ventral articular facet, which was compressing on the spinal cord, attributed to its fixed and malaligned position within the atlantooccipital joint. Following facetectomy, the stabilization of the occipital bone to the C2 vertebra was achieved by screws, wire, and polymethyl methacrylate. Two days after surgery, the dog recovered ambulation and showed gradual improvement in gait, despite mild residual ataxia. Postoperative CT and radiographs showed successful decompression of the spinal cord. The screw loosening was confirmed at 114 days, which was managed successfully by extracting the affected screws. Through the 21-month monitoring period, the dog showed a normal gait with a wide-based stance of the pelvic limbs when standing and experienced no pain. This case represents the first report of concurrent congenital AOD and AAI treated with a ventral surgical approach, contributing new insights to the understanding and management of such complex cranio-cervical junction disorders in veterinary neurosurgery.

## 1. Introduction

Atlanto-occipital dislocation (AOD) is defined as a critical and infrequent disorder at the cranio-cervical junction, characterized by the dislocation of the occiput from the vertebral column. It mainly results from abnormalities in ligamentous structures, such as the apical ligament and alar ligaments [1,2]. This condition, though rare in dogs and cats, emerges due to the failure of the inherent stability mechanisms at the cranio-cervical junction [3]. AOD frequently coexists with other cranio-cervical junction anomalies (CCJAs). This association could be explained by embryology, as the occipital bone and the first two cervical vertebrae develop together, incorporating the sclerotomal occipital region into the skull and integrating the cranial part of the spinal cord into the brain. CCJAs include atlanto-axial instability (AAI), atlanto-occipital overlapping (AOO), and Chiari-like malformation [4,5,6,7]. In both veterinary and human medicine, traumatic events are the primary cause of AOD, resulting in significant dynamic instability between the atlanto-occipital (AO) joint. However, in humans, it can also emerge in conjunction with conditions such as rheumatoid arthritis, Down syndrome, congenital cervical vertebral fusion, and neoplasia [1,3,8,9,10]. The paucity of reported cases is potentially due to the rapid progression towards mortality and diagnostic challenges within the veterinary field [11,12].

The clinical manifestation of AOD encompasses a range of neurological deficits, from acute cervical discomfort to pronounced ataxia and tetraparesis, and poor to grave prognosis [13]. The dislocation can also compress the spinal cord and brainstem at the foramen magnum, resulting in acute respiratory failure [14]. Diagnostic protocols begin with comprehensive neurological evaluations and are followed by lateral cervical spine radiographs to identify the location of the neurological lesion [15]. Displacement of the atlas with widening of the joint space on lateral view of the cervical spine can be an indication for an advanced imaging [13]. Various radiological evaluations using computed tomography (CT), such as the Traynelis classification, Powers’ ratio, the X-line method, and the Harris method, have been utilized in human medicine. However, the absence of established radiological criteria for AOD in veterinary medicine necessitates the use of advanced imaging modalities, such as magnetic resonance imaging (MRI) [10]. MRI scans precisely delineate the extent of ligamentous damage and assess the severity of associated spinal cord and brainstem injuries [15].

In human medicine, the treatment of AOD has evolved to include both conservative management and surgical intervention, depending on the severity of the dislocation and the presence of associated injuries. Conservative treatment often entails external coaptation to immobilize the cervical region, and surgical options include decompressive procedures such as dorsal laminectomy and partial occipital bone removal above the foramen magnum [3,16,17]. Internal fixation techniques, employing a variety of materials such as cortical screws and interarticular wires, aim to stabilize the cranio-cervical junction, thus mitigating the risk of further neurological deterioration [3,16,17,18].

To the best of author’s knowledge, though there are a few reported cases of traumatic AOD in dogs that have been corrected through ventral fixation, there is no single case of congenital AOD with concurrence of AAI in a dog. This case report aims to describe a novel surgical approach to the treatment of a dog diagnosed with concurrent congenital AOD and AAI. Despite the limited number of documented cases involving both conditions in the veterinary literature, this report highlights the successful application of a C1 vertebra facetectomy and ventral fixation of the occipitoatlantoaxial (OAA) complex.

## 2. Case Description

**Figure 1:**
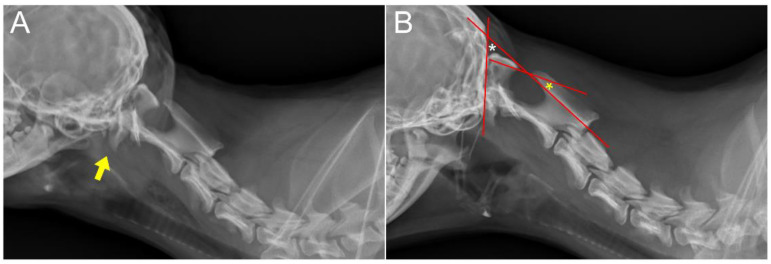
Pre-operative radiographs. (**A**) Lateral extension view of the cervical region. C1 is overlapping on the dorsal aspect of the occipital bone (arrow). (**B**) Dogs suspected of having AAI can be diagnosed by positioning their necks at 51° ± 10° of flexion (white asterisk). An atlas to axis angle (AAA) of 10° or greater (yellow asterisk) in this position can be indicative of AAI.

**Figure 2:**
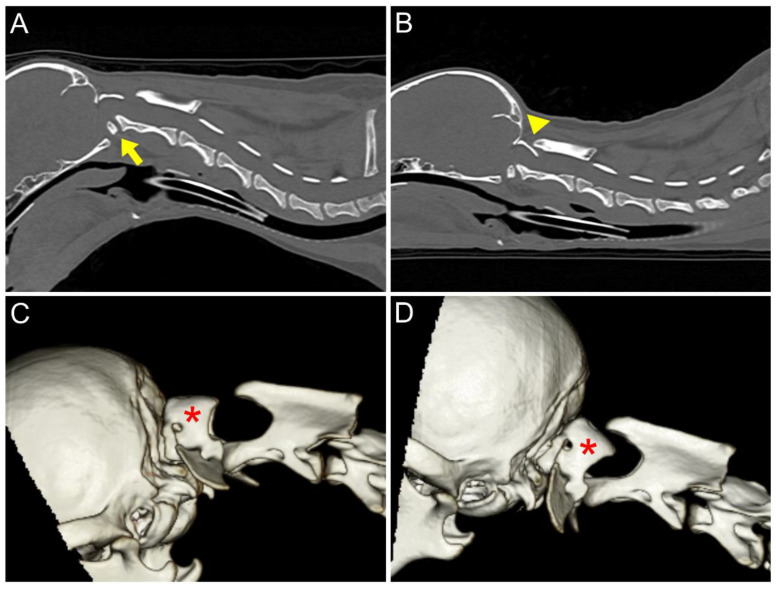
Pre-operative computed tomography. (**A**) Sagittal flexion view of the cervical region. Anomaly of ventral part of C1 (arrow). The ventral compression was caused by the ventral articular surface at the OAA during flexion. (**B**) Sagittal extension view of the cervical region. The C1 lamina is intruding into the spinal canal (arrowhead). The dorsal compression was due to the C1–C2 junction and the atlas dorsal lamina during extension. (**C**,**D**) Three-dimensional reconstructed CT image of the cervical region showed that regardless of whether the position was in flexion or extension, the atlas was rotated compared to the basioccipital bone (red asterisk).

**Figure 3:**
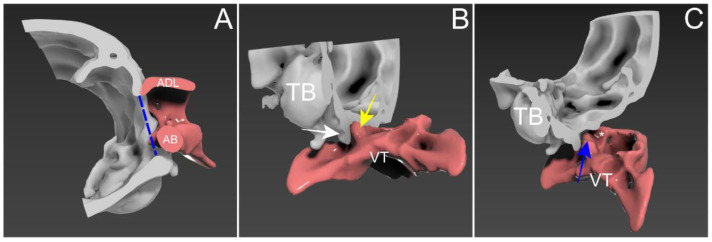
Three-dimensional reconstruction of caudal cranium and atlas using computer tomography. (**A**) Mid-sagittal view of the occiput–atlas interface. The atlas body (AB) and atlas dorsal lamina (ADL) are dislocated dorsally. The AB has moved into the foramen magnum level (blue dashed line), and the ADL impinges on the ventral border of the supraoccipital bone. (**B**) A parasagittal view at the level of the occipital condyles and the atlas in ventrodorsal projection. An atlas facet (yellow arrow) is dislocated dorsally over the occipital condyle (white arrow), indicating a disarticulated atlanto-occipital joint. (**C**) Similar projection with further clockwise rotation. Atlas was moved caudally to confirm the articular surface. The true cranial articular fovea (blue arrow) shows hypoplasia, and a falsely formed fovea (asterisk) was noticeable. TB, tympanic bulla; VT, atlas ventral tubercle.

**Figure 4:**
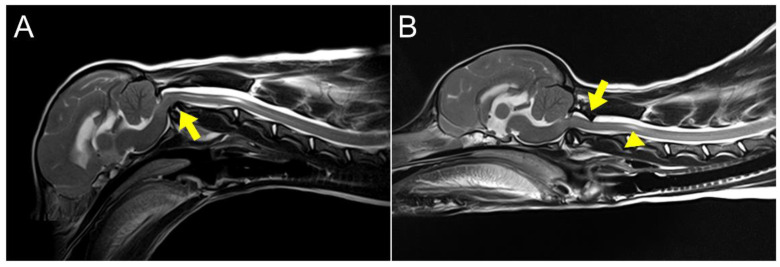
Dynamic magnetic resonance imaging. (**A**) Sagittal flexion view of the cervical region. Dens compressing the spinal cord in the C1~C2 region (arrow). (**B**) Sagittal extension view of the cervical region. Rotation of the atlas body backward resulted in compression on the ventral aspect of the medulla oblongata, and the atlas lamina compressed the spinal cord dorsally (arrow). As a result, hydromyelia and accumulation of cerebrospinal fluid (CSF) in the subarachnoid space were also observed (arrowhead).

An 8-month-old, 3.4 kg, castrated male Toy Poodle was referred due to a four-month history of non-ambulatory tetraparesis, with no traumatic event. The owner described an ataxic gait, which progressed to tetraparesis, starting when the dog was two months old. Physical examination revealed respiratory distress and cyanosis, along with non-ambulatory tetraparesis. These clinical signs and history could lead to the conclusion that the injury was chronic. The neurological status was assessed to be a grade 3 according to modified Frankel score [19]. Neurological examination revealed delayed proprioception, postural reactions, and mild exaggerated spinal reflex in all four limbs, with no abnormalities detected in cranial nerve function. Radiographic examination revealed luxation between the C1 and C2 vertebra, along with dislocation of the occipital bone and C1 (Figure 1A,B). AAI was objectively diagnosed by applying a 51° flexion cervical radiography [20] (Figure 1B). For the further assessment, two series of MRI and CT, with the neck in different positions with both extension and flexion, which could be defined as dynamic CT and MRI [21,22], were conducted. However, the neck’s range of motion was limited due to the instability and risk of deterioration. The patient was premedicated with butorphanol (0.2 mg/kg IV). Induction was achieved with propofol (5 mg/kg IV) and maintained with isoflurane (end-tidal concentration 1.1–1.6). The flexion view was captured in dorsal recumbency, and the extension view was taken in ventral recumbency (Figure 2). The CT findings revealed dorsal dislocation of the atlas from the occipital bone, along with luxation between the C1 and C2 vertebrae, accompanied by hypoplasia of bilateral occipital condyles, wings of atlas, and dens (Figure 2). The disarticulation of the AO joint could be diagnosed as AOD. The diagnosis was enhanced by the 3D reconstructed models (Figure 3). Identifying ligamentous abnormalities associated with AOD through subsequent MRI was challenging in this case. A dynamic MRI, performed in the identical position as the CT scan, showed that a disarticulated C1 caused dorsal compression of the spinal cord between C1 and C2 in the sagittal plane during extension, resulting in an enlarged dorsal subarachnoid space. Additionally, AAI, along with mild dens hypoplasia, led to ventral compression of the medulla oblongata and cranial spinal cord in the sagittal plane during flexion. This compression was responsible for the spinal cord injury, characterized by medullary kinking (Figure 4). These imaging studies have shown that in the flexion view, ventral compression of the spinal cord is more distinctly observed, whereas in the extension view, dorsal compression becomes more evident. The dog was diagnosed with concurrent AAI and AOD based on the findings from a 3D reconstructed CT image (Xelis, INFINITT Healthcare Co., Ltd., Seoul, Republic of Korea) (Figure 2C,D). The surgical plan involved performing ventral fixation for primary stabilization from the occipital bone to the C2 using screws, wires, and polymethyl methacrylate (PMMA). Two days before the surgery, the C-reactive protein (CRP) level had risen to 8.7 mg/L (reference range 0.1–1 mg/L), and the white blood cell (WBC) count was also elevated to 39,810/μL (reference rage 5050–16,760/μL).

Dexamethasone (0.2 mg/kg IV), an anti-inflammatory agent, was injected prior to the anesthesia. Induction of anesthesia was achieved with midazolam (0.2 mg/kg IV) and alfaxalone (3 mg/kg IV), and inhalation anesthesia was maintained with isoflurane (end-tidal concentration 1.4–1.9) in oxygen 100% [23]. Intraoperative analgesia was provided by a constant rate infusion (CRI) of remifentanil (0.1–0.3 μg/kg/min). Amoxicillin and clavulanic acid (AMC) (12.5 mg/kg IV) were administered every 120 min, with the first dose given 30 min before the incision. Anesthesia was continuously monitored using an anesthesia monitoring machine, which tracked heart rate, respiratory rate (RR), end-tidal CO_2_ (EtCO_2_), arterial oxygen saturation (SpO_2_), non-invasive blood pressure (NIBP), and esophageal temperatures. End-tidal isoflurane concentrations were also measured throughout the procedure. The dog was placed in dorsal recumbency with a vacuum sandbag to maintain neck extension and extension of the thoracic limbs caudally [24,25]. Typically, the neck is immobilized in an extended position during AAI surgery, but in this case, positional reduction with slight flexion of the neck was performed, as dorsal spinal cord compression by the C1 lamina was confirmed in the extended position during MRI.

**Figure 5:**
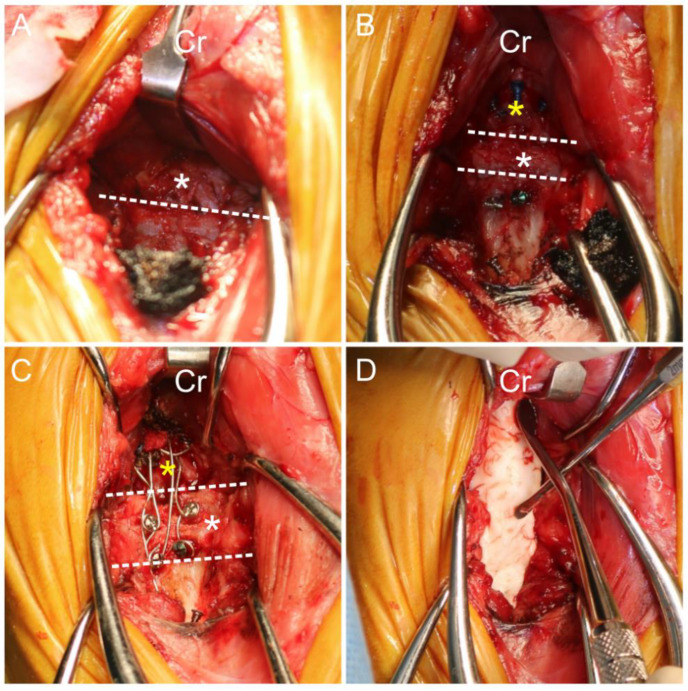
Intraoperative images. (**A**) Paramedian incision was employed for access, with meticulous removal of ventral fibrous tissue from the occipital (yellow asterisk), C1 (white asterisk), and C2 regions. (**B**) Screws were installed for stabilization following C1 vertebra facetectomy. (**C**) Wire was installed. (**D**) PMMA was installed for additional stabilization. Cr, cranial; dashed line, boundary between the vertebrae.

A ventral paramedian incision [26] was performed to approach the occipital, C1, and C2 vertebrae. Despite the complete removal of fibrous tissue on the ventral side and attempted reduction, realigning the atlanto-occipital joint to its normal position was challenging due to excessive soft tissue fibrosis, especially on the dorsal side. As an alternative, medullary decompression was achieved by partially removing parts of the basioccipital and ventral surface of the articular facet of the atlas using an electric high-speed drill and Kerrison rongeur (Figure 5). When the ventral atlanto-occipital membrane was opened, the distinct structures of the alar and apical ligaments could not be identified. Considering the dynamic CT measurements, where the angle between the basioccipital axis and the C2 vertebral body axis was 167° during extension and 132° during flexion, we performed positional reduction intraoperatively using fluoroscopy to maintain an angle within this range. Following this, 1.4 mm self-drilling screws (LeForte system, Jeil Medical, Seoul, South Korea) were used as anchors for PMMA and wire. The order of insertion was the occipital bone, axis, and atlas. Specifically, three 6 mm screws were placed into the occipital condyles of the occipital bone, two 8 mm screws were inserted into the pedicles of C1, two 6 mm screws were placed towards the cranial articular surfaces of C2, and two 8 mm screws were inserted into the caudal vertebral body of C2 [27]. The screws were augmented with 0.46 mm flexible orthopedic wire. An autogenous cancellous bone graft from the left humeral greater tubercle was applied into each joint space of the OAA interface for bony fusion. The OAA interface was then stabilized with PMMA. A gastrostomy tube was placed for dietary management. Respiration was maintained with mechanical ventilation following the incision. Spontaneous breathing was suppressed by increasing tidal volume, ensuring it did not exceed a peak airway pressure of 20 cmH_2_O. The pressure control ventilation mode was used with an inspiration pressure of 13 mmHg, respiratory rate adjusted between 9 and 13 breaths per minute based on ventilation needs, and an inspiratory-to-expiratory ratio set at 1:2. Spontaneous breathing was recovered as anesthesia was reduced towards the end of the surgery. Although hypotension occurred during the procedure, it was effectively managed with the early administration of dobutamine (5 μg/kg/min CRI), maintaining stable blood pressure throughout the operation.

**Figure 6:**
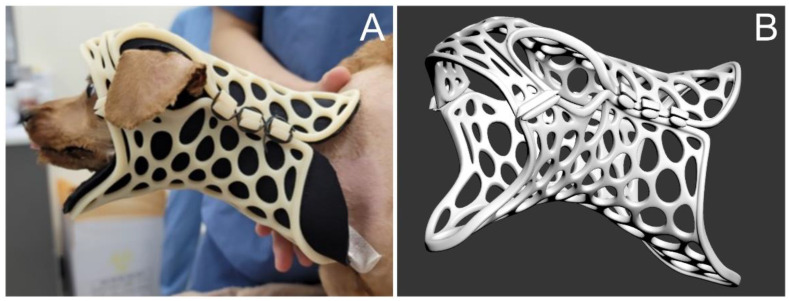
Three-dimensional-printed neck brace. (**A**) The patient wearing the 3D-printed neck brace. (**B**) Three-dimensional modeling of the neck brace using the program (3ds Max, Autodesk, San Francisco, CA, USA).

Postoperatively, remifentanil was administered for analgesia via a CRI of 0.1–0.3 μg/kg/min for three days, which was titrated to effect according to the Colorado State University Canine Acute Pain Scale (CSU-CAP) [28]. The analgesic opioid was converted to fentanyl transdermal patches (12 μg/h) for easing patient care and movement, which was maintained for an additional three days. To relieve neuropathic pain, pregabalin (4 mg/kg orally, q12h) was prescribed for 2 weeks and then tapered to 2 mg/kg q12h until postoperative day (POD) 19. Prednisolone at a dose of 0.5 mg/kg every 12 h was administered orally for seven days post-surgery. The dosage was then tapered to 0.375 mg/kg until the patient was discharged. After discharge, the dosage was further reduced to 0.25 mg/kg for one week before discontinuation. AMC (12.5 mg/kg, q12h) was administered as postoperative antibiotics for seven days. A 3D-printed neck brace was applied for external stabilization for 70 days (Figure 6). By three days postoperatively, CRP was 4.3 mg/L, and WBC was 32,990/μL, but these parameters had returned to normal by the time of discharge. Hematocrit was decreased from 43.2% (two days before the surgery) to 27.5% (reference range 37.3–61.7%) by three days postoperatively, then rebounded within reference range by discharge.

**Figure 7:**
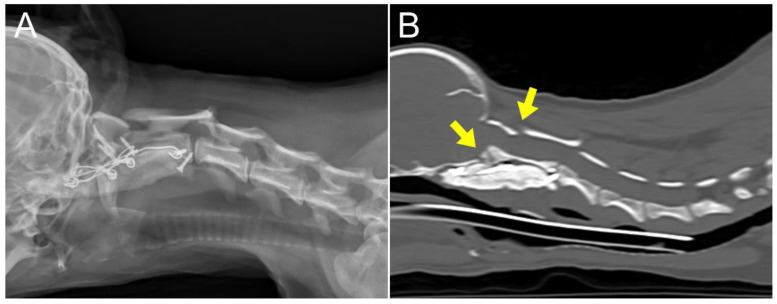
(**A**,**B**) Six days postoperative radiographs and computed tomographic assessments indicated the elimination of the previously observed spinal cord compression (arrow).

The patient recovered ambulation 2 days after surgery and showed gradual gait improvement with mild thoracic limb hypermetria. During the convalescence period, neurological examinations were performed at least every 12 h to monitor any neurological deterioration, such as cranial nerve dysfunction, especially pharyngeal and laryngeal dysfunction [29]. At six days postoperatively, radiographs and CT scan were performed to confirm the decompression of the medulla oblongata and cranial spinal cord, under sedation (Medetomidine, 30 μg/kg IM). The CT scan with extension view showed improvement; the portion of the C1 lamina compressing the dorsal aspect of the spinal cord was aligned parallel to the C1 dorsal tubercle and the C2 spinous process (Figure 7). The patient was discharged 19 days postoperatively, following owner education, without any postoperative complications. At discharge, the patient was evaluated as an improved grade 5 on the modified Frankel score from an initial grade 3 [19]. At two months postoperatively, radiographs revealed no pathological findings. Though hypermetria had improved gradually, it remained.

**Figure 8:**
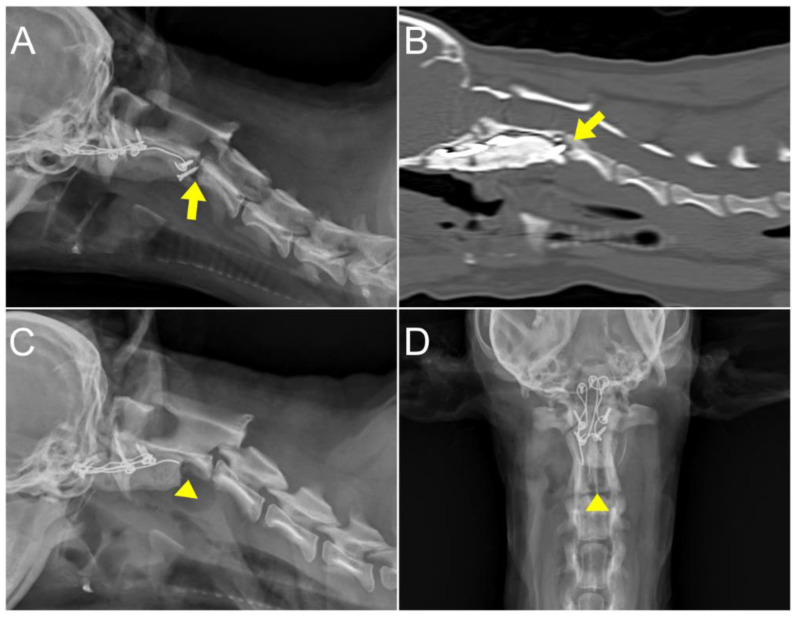
Post-operative radiographic and CT images 4 months after the surgery. (**A**,**B**) Radiographs and CT examinations revealed a rounded alteration in bone density surrounding the screw adjacent to the caudal endplate of the axis (arrow). (**C**,**D**) A revision surgery was performed to remove the loosened screws and PMMA (arrowhead).

At 4 months postoperatively, the dog exhibited unexplained cervical pain unresponsive to medication (gabapentin, 10 mg/kg q12h), prescribed at the primary veterinarian. An elevated CRP level (8 mg/L) with normal WBC level was found, prompting further radiographic and CT examination. These images revealed decreased bone density around the screw located near the caudal endplate of C2 (Figure 8). The loosened screws and surrounded PMMA were removed through revision surgery, administering AMC (12.5 mg/kg IV) every 120 min prophylactically, with the first dose given 30 min before the incision. The culture from the removed screw indicated septic loosening due to an Escherichia coli infection. Empirical administration of AMC (12.5 mg/kg IV q12h) and clindamycin (11 mg/kg IV q24h) was initiated from POD 1 until POD 5. Enrofloxacin (5 mg/kg IM q24h) was added from POD 5 for 14 days, depending on the results of antibiotic sensitivity tests. Pain management, depending on the patient status of CSU-CAP, was similar to the first surgery, with administration of remifentanil from the surgery to POD 2, and this was followed by fentanyl transdermal patches from POD 3. Additional pain control was achieved with gabapentin at 10 mg/kg, q12h for 18 days. This regimen led to pain relief and a decrease in CRP levels. The patient was discharged 11 days after surgery without any postoperative complications. Subsequent radiography and physical examination revealed no abnormalities until 12 months postoperatively. The patient continued to be monitored until 21 months postoperatively; during this time, no signs of pain were noted, and there was an improvement in gait, which was close to normal, although the pelvic limbs displayed a slightly wide-based stance while standing.

## 3. Discussion

To the best of the authors’ acknowledge, AOD has been rarely described in the veterinary literature, and the extent of research has been limited to traumatic AOD [3,13,17,18,30,31]. Historically, treatments have ranged from conservative approaches like closed reduction to more invasive procedures such as open reduction and internal fixation [12,13,16,30]. Both ventral and dorsal stabilization techniques have been reported for an acute trauma patient, with some cases also undergoing dorsal laminectomy [17,18,31,32]. This particular case is significant, as it represents the first veterinary instance where ventral facetectomy was combined with ventral stabilization of the O-C1-C2 complex, leading to successful management over 21 months [18,33,34]. This was despite the patient’s condition being so severe and chronic that realignment of the atlas and occipital bones was considered unfeasible.

This case represents a unique instance of a chronic and congenital form of AOD that was distinct from AOO. In this case, the diagnosis of AOD could be confirmed by identifying the disarticulation of the AO joint, which was suspected to result from congenital abnormalities in the OAA complex, through advanced imaging. Based on the 3D reconstructed models, this dog exhibited the facet of the atlas being dorsally dislocated over the occipital condyle. The dorsal lamina of the atlas impinged on the ventral border of the supraoccipital bone without overlapping into the foramen magnum. Considering that AOO is characterized by the atlas shifting craniodorsally and overlapping the occipital bone without disarticulation of the joint, this distinction clarified the differentiation of AOD from AOO (Figure 3) [35]. Although MRI findings of abnormalities in ligamentous structures have been described in the human literature [36], identifying those associated with AOD was challenging in this case. However, we could confirm ventral ligamentous abnormalities were present during the surgery. Evidence for congenital malformation in this patient included the early onset of symptoms, morphological alterations in the AO joint interface on CT, and the absence of any traumatic event. There may be a possibility of microdamage from instability, which likely contributed to this condition.

The presence of a significant amount of fibrotic scar tissue surrounding the joints and adjacent soft tissues, attributed to chronic factors, caused limited joint motion and impeded the approach to the surgical site and reduction of the dislocation [9,31]. During the ventral approach, it was challenging to remove all the fibrotic scar tissue, especially that on the dorsal side, to facilitate atlanto-occipital realignment. Excessive tissue removal was avoided to prevent further joint instability, as too much removal could exacerbate the instability [18,31]. In this case, despite meticulous removal of the fibrotic tissue and incision of the ventral atlantooccipital membrane, complete anatomical reduction could not be achieved.

To overcome this incomplete anatomical reduction, we planned to address the dynamic compression lesions on both sides. Ventral compression was primarily caused by the ventral articular surface at the OAA during flexion, and the dorsal compression was caused by the C1–C2 junction and the atlas dorsal lamina during extension. To resolve the ventral compression, the reduction of the AO joint and realignment of the vertebral column was thought to be the best treatment, but the complete reduction was not possible. Therefore, we performed decompression by a partial facetectomy of the atlas and a basioccipital partial craniectomy. However, the removal of joint structures increased the instability of the cranio-cervical junction. For comprehensive stabilization, we modified the conventional screw and PMMA fixation technique by placing an orthopedic wire between the screws to provide compression and augmentation [37,38,39].

For the resolution of dorsal compression, we opted for positional reduction based on dynamic MRI findings. We initiated the multiple joint (OAA) arthrodesis with the patient in a neutral position with a slight flexion, where compression would potentially be less severe. Notably, all procedures were carried out through a single ventral incision, which reduced surgical time and minimized potential complications associated with multiple incisions. Furthermore, previous studies have indicated a higher success rate with ventral fixation compared to dorsal fixation in surgically treating AAI [24]. Alternatively, ventral fixation with dorsal decompression via atlas dorsal laminectomy could effectively address the compression under a neck extension position. However, there would be comorbidity from a double incision and instability due to the removal of dorsal atlantoaxial ligament and membrane. In this particular case, the ventral approach and fixation effectively addressed both ventral and dorsal compressions and enhanced the stability of the OAA interface.

Comprehensive evaluations of the cranio-cervical junction can be conducted by dynamic advanced imaging with the head and neck in various positions, such as mild flexion and mild extension [4,5,35]. Through dynamic MRI, we confirmed dynamic compressions on both the dorsal and ventral aspects of the cervical spinal cord. These compressions were identified as potential sources of neurological signs, such as neuropathic pain and ataxia, which occur when the neck is moved. The degree of compression was relatively lower in a flexed position compared to an extended state in this case. Therefore, planning appropriate surgical techniques based on dynamic imaging findings is recommended for these conditions. Consequently, for dogs having AOD and AAI concurrently, the utilization of dynamic advanced imaging techniques could be a valuable tool for evaluating these conditions and formulating treatment plans.

The differential diagnosis of septic screw loosening included surgical site infection (SSI) and hematogenous infection. Several studies described the occurrence of SSI related to peri-implant infection up to one-year post-surgery [40]. Postoperative antibiotic use may contribute to the late onset of peri-implant infection. When antibiotics are administered, bacteria growth may be suppressed, but after the medication is withdrawn, the bacteria might proliferate. Furthermore, PMMA is known to be more susceptible to infection [41]. The patient had no pre-existing infectious diseases, such as pyoderma, bacterial cystitis, and periodontitis, which are the most common sources of hematogenous infection [42]. Consequently, we assumed that SSI was the source of the septic loosening.

This case report has several limitations. First, it is based on a single patient and has limitations of the single-case nature. We were unable to compare between various surgical techniques introduced in diverse literature and our technique. Moreover, the inability to evaluate the degree of spinal cord parenchymal compression using dynamic advanced imaging from multiple angles restricted the evaluation. Therefore, the best position for assessing AOD was not elucidated. Finally, this report does not assess whether a dorsal approach could have allowed for more accurate reduction and stabilization than the ventral approach used, adding to the limitations in evaluating the best surgical orientation for cases like this.

## 4. Conclusions

In concurrence of AOD and AAI, dynamic CT and MRI serve as the pivotal tools for diagnosing and establishing a surgical plan. This plan might involve surgical decompression using ventral placement of screws and PMMA, which has been effective for the single case presented here. In instances where reduction poses challenges, implementing ventral facetectomy can significantly aid decompression by alleviating areas of cord compression and stabilizing the occipitoatlantoaxial joint.

## Data Availability

The original contributions presented in the study are included in the article. Further inquiries can be directed to the corresponding author.
